# Inhibition of colony stimulating factor-1 receptor (CSF-1R) as a potential therapeutic strategy for neurodegenerative diseases: opportunities and challenges

**DOI:** 10.1007/s00018-022-04225-1

**Published:** 2022-04-02

**Authors:** Jinming Han, Violeta Chitu, E. Richard Stanley, Zbigniew K. Wszolek, Virginija Danylaité Karrenbauer, Robert A. Harris

**Affiliations:** 1 Department of Neurology, Xuanwu Hospital, Capital Medical University, Beijing, China; 2 Department of Developmental and Molecular Biology, Albert Einstein College of Medicine, Bronx, NY 10461 USA; 3 Department of Neurology, Mayo Clinic, Jacksonville, USA; 4 Department of Clinical Neuroscience, Center for Molecular Medicine L8:04, Karolinska Institutet, Karolinska University Hospital, 171 76 Stockholm, Sweden; 5 Department of Neurology, Karolinska University Hospital, Stockholm, Sweden; 6 Applied Immunology and Immunotherapy, Department of Clinical Neuroscience, Center for Molecular Medicine, Karolinska Institutet, Karolinska University Hospital, Solna, Sweden

**Keywords:** Colony stimulating factor-1 receptor, Alzheimer’s disease, Parkinson’s disease, Huntington’s disease, Amyotrophic lateral sclerosis, Multiple sclerosis

## Abstract

Microglia are specialized dynamic immune cells in the central nervous system (CNS) that plays a crucial role in brain homeostasis and in disease states. Persistent neuroinflammation is considered a hallmark of many neurodegenerative diseases, including Alzheimer’s disease (AD), Parkinson's disease (PD), Huntington’s disease (HD), amyotrophic lateral sclerosis (ALS) and primary progressive multiple sclerosis (MS). Colony stimulating factor 1-receptor (CSF-1R) is predominantly expressed on microglia and its expression is significantly increased in neurodegenerative diseases. Cumulative findings have indicated that CSF-1R inhibitors can have beneficial effects in preclinical neurodegenerative disease models. Research using CSF-1R inhibitors has now been extended into non-human primates and humans. This review article summarizes the most recent advances using CSF-1R inhibitors in different neurodegenerative conditions including AD, PD, HD, ALS and MS. Potential challenges for translating these findings into clinical practice are presented.

## Background

Microglia are the predominant resident immune cells of the central nervous system (CNS), deriving from yolk sac progenitors during early neurodevelopment [1–4]. Under steady-state conditions, they actively contribute to myelinogenesis [5] and synaptic pruning [6]. Upon detection of non-homeostatic disturbances microglia become rapidly activated and proliferate. They develop into a broad range of activation states depending on the disease stage [7–10] and microenvironment [11–13]. It is generally accepted that initial activation of microglia may exert beneficial effects on disease recovery by phagocytosing cellular and myelin debris and favoring remyelination which in turn is thought to limit axonal dysfunction and loss [14]. In contrast, prolonged microglial activation may contribute to chronic neuronal damage and impede regeneration [15–17].

Emerging data suggest that microglial activation is a hallmark of a number of neurodegenerative diseases, including Alzheimer's disease (AD) [18, 19], Parkinson's disease (PD) [20], Huntington’s disease (HD) [21], amyotrophic lateral sclerosis (ALS) [22, 23] and primary progressive multiple sclerosis (MS) [24, 25]. Furthermore, a variety of genes identified as risk factors for neurodegenerative diseases are expressed in microglia [26]. Developing strategies for replacing defective microglia or modulating microglial function may therefore be a novel approach to treat neurodegenerative diseases [27–29]. As our understanding is rapidly evolving, we herein focus on the most updated preclinical and clinical evidence regarding potential microglia-based therapy in neurodegenerative diseases through targeting of the colony stimulating factor-1 receptor (CSF-1R).

## The CSF-R and its ligands, CSF-1 and IL-34

CSF-1R is a receptor tyrosine kinase belonging to the platelet-derived growth factor receptor (PDGFR) family [30]. CSF-1R can be activated by two different homodimeric ligands, CSF-1 (also known as macrophage-colony stimulating factor, M-CSF) and interleukin-34 (IL-34) which have limited (~ 10%) primary sequence homology but share a similar three-dimensional structure and bind the same site on the CSF-1R [30]. While CSF-1R appears to be the sole receptor for CSF-1, IL-34 also binds receptor protein-tyrosine phosphatase-ζ (RPTP-ζ) [31]. The ligands also differ in their pattern of expression. Within the central nervous system (CNS), CSF-1 is predominantly expressed in the corpus callosum, cerebellum and areas of the olfactory bulb, cortex and hippocampus, while IL-34 is expressed throughout the forebrain but at very low levels in the cerebellum [32–34]. Physiologically, CSF-1 is essential for embryonic microglial development, while IL-34 is mainly involved in their post-natal development and maintenance [35, 36]. CSF-1 and IL-34 may regulate the development and maintenance of different subpopulations of microglia. In mice, neither developmental genetic targeting nor function blocking antibodies to either CSF-1 or IL-34 cause a complete loss of brain microglia [32–34, 37, 38]. Instead, they result in a region-specific loss that corresponds to the complementary expression patterns of each ligand [39]. Interestingly, CSF-1R signaling and the expression of CSF-1 can be significantly upregulated during inflammatory conditions [16, 40], with CSF-1 expression being upregulated in disease-associated microglia [7] and possibly contributing, in an autocrine fashion, to their expansion.

**Fig. 1:**
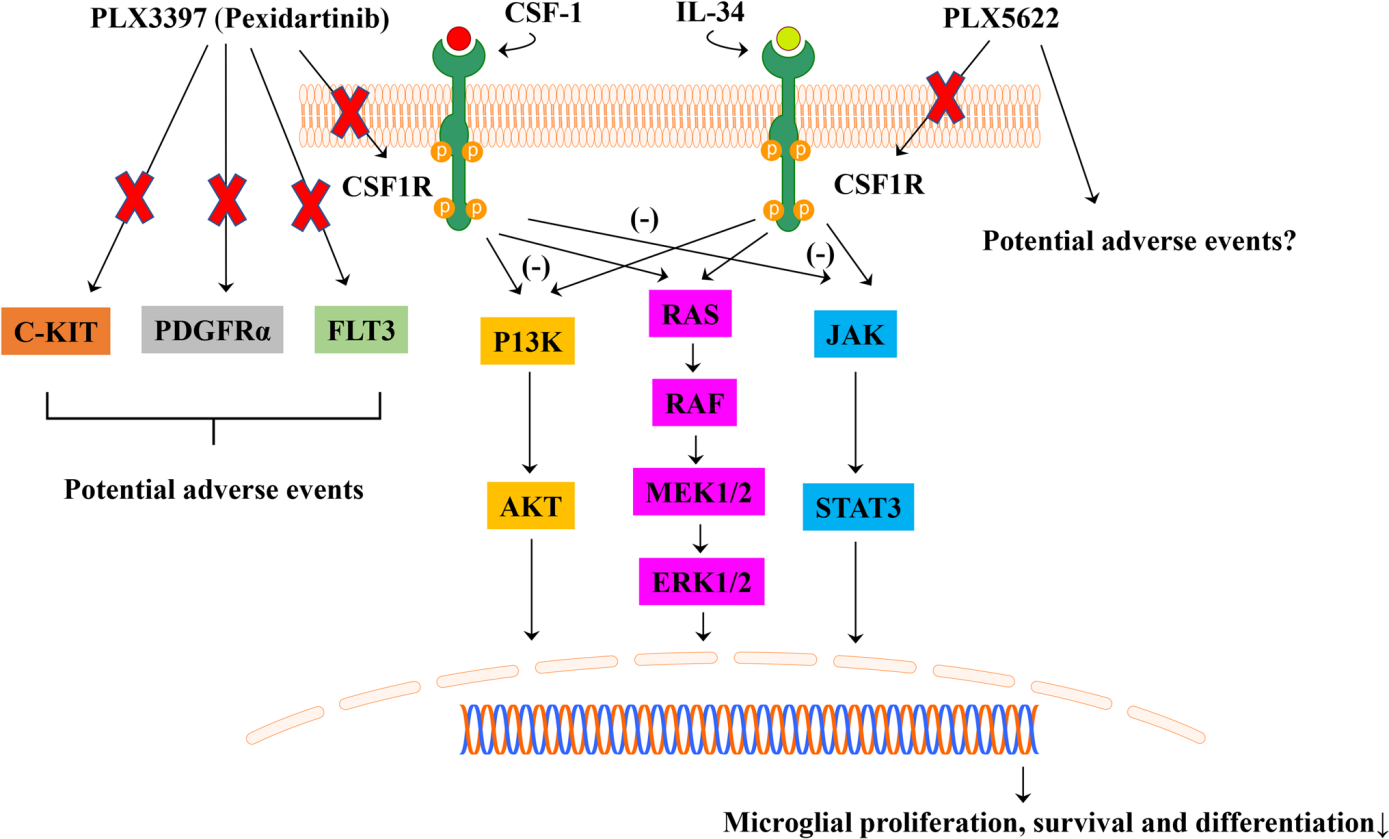
CSF-1R signaling pathways and the effects of CSF-1R inhibitors. CSF-1 and IL-34 share a common receptor, CSF-1R. After binding to the CSF-1R, a cascade of downstream signaling molecules is activated, including those involved in the PI3K-AKT, ERK1/2 and JAK/STAT signaling pathways, promoting cellular proliferation, survival and differentiation. PLX3397 (Pexidartinib) and PLX5622 are the most widely used CSF-1R inhibitors, with favorable tolerability profiles. Treatment with PLX3397, or PLX5622, causes effective depletion of microglia. PLX3397 also inhibits C-KIT, PDGFRα and FLT3. Consequently, in clinical practice, the broader effects of PLX3397 may cause adverse effects, including hair discoloration and hepatotoxicity. PLX5622 is a novel CSF-1R inhibitor with a higher selectivity. (-) indicate inhibitor-induced reduction in signaling through the respective pathways

Structurally, the CSF-1R comprises five extracellular immunoglobulin domains (D1–D5), a transmembrane domain and an intracellular split kinase domain [30]. Ligand binding triggers CSF-1R autophosphorylation and induces a cascade of downstream signaling events, which regulate cellular survival, proliferation, differentiation and motility [30, 35]. Among these, the phosphoinositide-3 kinase (PI3K)/Akt pathway plays an important role in regulating CSF-1R-mediated macrophage survival [30, 41, 42] (Fig. 1) and mediates signaling for cell viability downstream of CSF-1R in other cell types [43].

## Genetic targeting of the CSF-1R or treatment with CSF-1R inhibitors causes depletion of microglia

Within the CNS, the CSF-1R is mainly expressed on microglia and plays an important role in microglial development and steady-state maintenance [1, 44, 45]. Genetic ablation or loss of function of the CSF-1R causes depletion of microglia in mice [1], zebrafish and man [46], confirming that, regardless of species, the survival, maintenance and proliferation of microglia are critically dependent on CSF-1R. Consistent with this, microglia can be effectively ablated using CSF-1R kinase inhibitors [28, 45, 47]. Recently, several CSF-1R inhibitors, including PLX3397 (Pexidartinib; Plexxikon, Inc.) [48], PLX5622 (Plexxikon Inc.) [49], BLZ945 (Novartis) [50], sCSF-1R_inh_ (Sanofi) [16], Ki20227 [51, 52], JNJ-40346527 (Johnson & Johnson) [53, 54], ARRY-382 (Array BioPharma) [54] or GW2850 [55, 56] have been tested in preclinical studies and clinical trials for a variety of conditions. Among these, Pexidartinib and PLX5622 have been the most widely used in rodent model research [45, 57, 58], which has been extended into non-human primates [59]. Pexidartinib is orally bioavailable, brain-penetrant [60] and exhibits a favorable tolerability and safety profile in human studies [54, 61]. Apart from targeting CSF-1R and c-Kit, Pexidartinib (PLX3397) shows limited cross reactivity with other tyrosine kinases and has 10 ~ 100-fold selectivity for c-Kit and CSF1R over other related kinases, including PDGFRα and FLT3 [62, 63]. PLX3397 binds to the autoinhibited CSF-1R through direct interactions with juxtamembrane residues embedded in the ATP-binding pocket and prevents ATP and substrate binding [63]. Consistent with this, PLX3397 efficiently suppresses the proliferation of CSF-1-dependent microglia and macrophage cell lines in vitro [63] and its administration in vivo induces a rapid loss of microglia [45]. More selective CSF-1R inhibitors including PLX5622 and GW2580 (PLX6134) have also been used to eliminate microglia and CSF-1-dependent macrophages in various research settings [64, 65]. PLX5622 is a novel CSF-1R inhibitor with a higher selectivity and brain penetrance than PLX3397 (Fig. 1) (reviewed in [66]). PLX3397 and PLX5622 can be integrated into rodent chow diet at different concentrations without significantly affecting adult mice behavior or cognitive functions [45, 66, 67]. JTE-952 is a highly specific and orally available CSF-1R inhibitor, with a relatively higher efficacy than GW2580 [40, 68].

We have previously demonstrated that approximately 95% of CD11b^+^CD45^low^Ly6C^−^Ly6G^−^ microglia can be effectively depleted after 21 days of PLX3397 treatment at a concentration of 290 mg/kg [69]. Withdrawal of these compounds in mice causes a rapid microglial repopulation solely derived from surviving resident microglia, rather than from Nestin^+^ stem cells [70]. Subsequently, there is an overshoot in repopulating microglial numbers, which become normalized to the baseline level after 3 weeks [71]. Other studies have indicated that both chronic and transient depletion of activated (senescent) microglia by CSF1R inhibitors may exert beneficial effects in aging by reducing inflammation, metabolic decline [49] and by reversing the changes in hippocampal neuronal complexity [71]. Importantly, the surviving resident microglia following CSF-1R inhibitor treatment have a remarkable capacity to regenerate the CNS and the newly repopulated microglia acquire a more homeostatic phenotype and can promote brain repair [27, 71, 72]. Thus, at least in mouse models of aging, CSF-1R inhibitors permit the elimination of activated microglia and their replacement by newly generated, more homeostatic microglia.

### Inhibiting the CSF-1R in AD

AD is the most common human neurodegenerative disease. Clinically, late-onset AD affects individuals over the age of 65 years, with the prevalence of early-onset patients being relatively low [73]. The hallmarks of AD pathology are the presence of extracellular amyloid beta (Aβ) and intraneuronal accumulation of fibrillar tangles of abnormally phosphorylated Tau (pTau) protein in the brain [74, 75]. The accumulation of insoluble and highly phosphorylated tau species that are assumed to be pathologically relevant can be detected by phosphorylation-dependent antibodies such as AT8 (phospho-Ser202 and phospho-Thr205), PHF1 (phospho-Ser396 and phospho-Ser404), and AT270 (phospho-Thr181) and the staging of AD cases is based on the staining of pTau Ser202/Thr205 with the specific antibody AT8 [76].

A widely used AD model is the 5xFAD mouse [77], in which the expression of 5 human familial AD disease mutations is driven by the *Thy1* promoter. In these mice, neuritic plaque deposition is evident at 2 months and neuronal loss starts from 10 months of age [57, 78–80]. Other models include the APP/PS1 transgenic mice expressing APP^Swe^ and PS1 mutations, the 3xTg-AD mice expressing the APP^Swe^, MAPT P301L and PSEN1 M146V transgenes. Models of AD tauopathy include the MAPT P301S mice, expressing the mutant form of human Tau, the TE4 knock-in mice, expressing Tau P301S and human ApoE4 and Tg4510 mice, expressing the P301L mutation. Studies in these models indicate that neuroinflammation plays an important role in the pathogenesis of AD, with microglia enacting a primary role [81–83]. In addition, resolution of inflammation, a tightly regulated process mediated by specialized pro-resolving lipid mediators to prevent over-responsiveness to tissue damage and infection, has been reported to be defective in AD patients [84, 85].

Mutations in *APOE* or *TREM2* genes expressed in microglia are strongly linked to an increased risk of developing AD [86–89]. Reactive/phagocytic microglia surrounding extracellular plaques promote Aβ plaque expansion [90]. In preclinical studies of AD, total or partial removal of microglia using CSF-1R inhibitors improved cognition (Table 1). Following CSF-1R inhibitor treatment, both a decrease [47, 58, 78, 91] and no change [57, 67, 92] in Aβ plaque load have been reported, respectively, and the effects on Tau pathology and phosphorylation were also variable. Microglial depletion at 4 or 6 months of age using PLX3397 caused a remarkable reduction of AT8 + pTau in two different tauopathy mouse models [93]. In another study, the same inhibitor attenuated the progression of pTau and halted brain atrophy in TE4 mice [94]. However, administration of a comparable dose of PLX3397 in older (12-month-old) Tg4510 mice produced only a 30% reduction in microglia numbers and failed to elicit changes in Tau pathology and phosphorylation [95]. Regardless of the extent of microglial depletion, or effects on Aβ and Tau, most studies agree that treatment with CSF-1R inhibitors decreases inflammation in AD [57, 67, 78, 91, 92]. Together, these data indicate that activated microglia, rather than Tau or Aβ direct neurotoxicity, mediate neurodegeneration in AD and suggest CSF-1R inhibitors as a potential therapeutic strategy in AD. However, since a small number of neurons, including mature forebrain cortical neurons and hippocampal neurons may express CSF-1R [96–98], the effects of CSF-1R inhibition on the accumulation of intraneuronal amyloid should also be investigated.

**Table 1:** Overview of preclinical studies using CSF1R inhibitors for the treatment of AD

AD models	CSF1R inhibitors	The extent of microglial depletion	Main outcomes	References
5xfAD mice (10 months old)	PLX3397 (290 mg/kg)	~ 80%	Improved hippocampal-dependent memory deficits Restored dendritic spine numbers Reduced inflammation-related gene expression Prevented neuronal loss	[57]
5xfAD mice (4 months old)	PLX5622 (1200 mg/kg)	Greater than 50%	Reduced plaque burden Reduced inflammatory transcripts and cytokines Enhanced neuritic dystrophy	[91]
5xfAD mice (2 months old)	PLX3397 (290 mg/kg)	~ 70–80%	Inhibited the accumulation of intraneuronal amyloid Inhibited the formation of neuritic plaques Improved behavioral performance Decreased the levels of pre-fibrillar oligomers in the plasma and brain	[78]
APP/PS1 mice (6–9 months old)	GW2580 (75 mg/kg)	Less than 50%	Inhibited abnormal microglial proliferation Prevented behavioral deficits Prevented synaptic degeneration Did not alter the levels of amyloid	[92]
3xTg-AD mice (15 months old)	PLX5622 (300 mg/kg)	~ 30%	Improved hippocampal-dependent memory deficits Prevented microglial association with plaques Did not alter the levels of plaque loads	[67]
APP/PS1 mice (12 months old)	PLX5622 (1200 mg/kg)	~ 70%	Reduced main elements of the leukotriene synthesis pathway Decreased mRNA levels of *Alox5* and *Alox5ap* Decreased mRNA levels of *Cysltr1* in the hippocampus and cortex	[83]
Tg4510 mice (12 months old)	PLX3397 (290 mg/kg)	~ 30%	Did not change Tau pathology and phosphorylation No significant changes of neuron loss after treatment No significant changes of blood vessel after treatment	[95]
P30IS mice (8 months old)	JNJ-40346527 (JNJ-527) (30 mg/kg)	~ 40%	Decreased the expression of proinflammatory cytokines Prevented motor neuron degeneration Reduced neuronal death	[53]
5xfAD mice (9 months old)	PLX3397 (50 mg/kg, oral gavage)	42%	Alleviated Aβ pathology in the cortex and hippocampus Increased the expression of synapse-related protein Rescued dopaminergic signaling	[47]
TE4 mice (6 months old)	PLX3397 (400 mg/kg)	∼100%	Rescued the brain volume loss Reduced ptau levels Recued soluble apoE level in TE4 mice	[94]
AAV-GFP/tau-injected mice (4 months) and PS19 mice (3.5 months)	PLX3397 (290 mg/kg)	86%	Inhibited tau propagation in the dentate gyrus	[93]
5xFAD mice (1.5 months)	PLX5622 (1200 mg/kg)	97–100%	Reduced plaque number and volume Prevented the downregulation of synaptic genes in the hippocampus	[58]

Interestingly, the effectiveness of microglial depletion following CSF-1R inhibitors in AD varies between studies and brain regions. Unlike the near complete ablation of microglia within a few days using PLX5622 in wild-type mice, microglial densities were depleted by only 30% in the subiculum and by 70% in the thalamus in 5xFAD mice [91]. Consistently, following PLX5562 treatment, deposited amyloid as assessed by immunostaining (6E10) was significantly decreased in the thalamus, where microglia were effectively removed, but not in the subiculum [91], suggesting that a near complete depletion of microglia following CSF-1R inhibitor treatment, but not a modest reduction, may exert beneficial effects in preclinical AD models (Table 1). Intriguingly, the presence of CSF-1R-inhibitor resistant microglia [99], associated with dense core plaques, was reported in the 5xFAD mouse model [57]. The factors contributing to the maintenance of these CSF-1R-inhibitor resistant microglia and their functional role in neurodegenerative diseases should be investigated.

### Inhibiting the CSF-1R in PD

After AD, PD is the second most prevalent neurodegenerative disease worldwide and it is characterized by progressive degeneration of dopaminergic neurons in the *substantia nigra* of the midbrain [100]. Most PD patients develop their clinical symptoms over the age of 60 and some cases can be caused by mutations in genes, including *PRKN*, *SNCA* and *LRRK2* [101, 102]. The significant loss of dopamine leads to classical idiopathic PD disease-like movement problems. Apart from typical clinical manifestations including rigidity, bradykinesia, resting tremors and postural instability, patients with PD also suffer from non-motor symptoms such as mood and sleep disorders [103]. PD is viewed as a multi-system disorder with complex mechanisms developing with disease progression. Levodopa is the gold-standard symptomatic treatment for PD. Most patients may experience motor complications after long-term treatment. Alternative potential therapeutic strategies to treat PD represent a current unmet medical need.

Neuroinflammation mediated by glial cells plays a pivotal role in the pathogenesis of PD and immune-targeted therapeutic strategies for treating PD are currently being tested [104]. Microglial functions are tightly controlled by neuronal activity and neurotransmitters secreted by healthy neurons [105, 106]. The death of dopaminergic neurons may cause microglia to lose their physiological functions and develop a pathological microglial phenotype [107]. The increased microglial activation measured by positron emission tomography (PET) during early PD disease stages has been reported to be inversely correlated with the loss of dopamine terminals [108]. Furthermore, persistent and uncontrolled stimuli, such as the accumulation of α-synuclein, also contribute to chronic neuroinflammation [109]. Microglia carrying PD genetic variants may fail to degrade cell debris, unfolded proteins and dying neurons due to endolysosomal impairments and dysfunctional phagocytosis [110]. Collectively, emerging evidence suggests that microglia can be targeted pharmacologically to prevent or delay PD [111, 112].

In a preclinical model of PD induced by stereotaxic injection of 6-hydroxydopa (6-OHDA), PLX3397 treatment at a concentration of 30 mg/kg initiated 7 days after the neurotoxic insult exerted a neuroprotective influence [113]. Specifically, both motor function and depressive-like behavior were alleviated following PLX3397 treatment, as measured by the adhesive removal test and forced swim test, respectively [113]. Strikingly, functional PET imaging demonstrated that PD rats treated with PLX3397 exhibited a lower uptake of radioactive translocator protein, a marker for neuroinflammation and glial activation, than did the PD group [113]. Although there are minor changes of dopamine transporters in PD rats following PLX3397 treatment, an increased tracer uptake was recorded in the treatment group as measured by dopaminergic and glutamatergic PET [113]. These results indicated that the therapeutic effects of CSF-1R inhibitors in PD can be achieved by reducing proinflammatory mediators. However, in another study of 4-phenyl-1,2,3,6-tetrahydropyridine (MPTP)-induced PD in mice, PLX3397-mediated depletion of microglia before the neurotoxic insult worsened locomotor performance and enhanced dopaminergic neurotoxicity and leukocyte infiltration, indicating a neuroprotective role of microglia in PD [62]. We propose that the differences in timing of microglial depletion (i.e. before, or after the injury of the dopaminergic system) might explain some of these described discrepancies.

The long-term safety and efficacy of CSF-1R inhibitors in PD remain to be further explored. If the dynamics of microglial responses during the initiation and progression in PD can be better understood, reactive microglia may then be targeted using CSF-1R inhibitor treatment within an appropriate time window.

### Inhibiting the CSF1-R in HD

HD is an autosomal dominant devastating neurodegenerative disorder resulting from the abnormal CAG trinucleotide expansion (36 repeats or more) in exon 1 of the huntingtin (*HTT*) gene, encoding a long polyglutamine tract of the huntingtin protein [114, 115]. The mean age at onset of typical HD symptoms is 30–50 years and disease onset is inversely correlated with the length of the CAG repeat [115]. Individuals with HD usually experience late-manifesting movement disorders, cognitive decline, behavioral abnormalities and psychiatric disturbances [115]. HD patient-reported symptoms include emotional issues, fatigue, difficulty thinking and daytime sleepiness [116]. It is well established that the degeneration of the striatum and widespread cortical atrophy with neuronal loss are hallmarks of HD brain pathology [117]. It is also important to note that neurodevelopment can be affected in the context of HD [118]. This finding was supported by abnormal HTT being mis-localized in the embryo, disrupting neuroepithelial junctional complexes and then shifting neurogenesis towards the neuronal lineage [118].

There are no effective treatments for HD, but modulation of immune activation to prevent or delay HD has recently attracted considerable attention [119, 120]. Neuroinflammation has a role in the pathogenesis of HD. Specifically, the presence of reactive microglia was noted in the striatum and cortex of HD human brains [117]. Furthermore, microglial activation was evident even before HD symptom onset, suggesting that these cells play an essential role in the progression of HD [121]. Indeed, neuronal mutant HTT (mHTT)-mediated neurotoxicity has been linked to increased numbers of microglia with a reactive phenotype, subsequently leading to cell death [117]. Cell-autonomous mechanisms induced by the intrinsic mutant protein also play a contributory role in HD-related microglial activation [122]. However, microglia have also been reported to suppress inflammation and to preserve neuronal function in the HD brain [117].

The role of CSF-1R-dependent microglia in HD has been explored in transgenic R6/2 mice expressing the human HTT gene containing more than 100 CAG repeats [123]. These mice experience progressive motor and behavioral abnormalities starting at 7 weeks of age. Increased Iba1^+^ microglial densities are evident in specific brain regions [123]. Sustained PLX3397 treatment at a concentration of 275 mg/kg starting at week 6 in R6/2 mice, significantly attenuated HD-related grip strength and object recognition deficits and normalized their dysregulated interferon gene signature [123]. Furthermore, PLX3397 treatment reduced the accumulation of mutant huntingtin (mHTT) by decreasing the numbers of intranuclear mHTT inclusion bodies in the striatum, without exerting significant influences on *HTT* gene expression. In addition, following sustained treatment, significant striatal volume loss was inhibited, independent of NeuN^+^ density changes and abnormal accumulation of chondroitin sulphate proteoglycans, primary components of glial scars, was significantly attenuated in the striatum, cortex and hippocampus [123].

Emerging data suggests that systemic administration of CSF-1R inhibitors at a relatively high dose exerts crucial influences on peripheral tissue macrophages [124, 125]. It is therefore possible that attenuation of HD disease-related behavioral functions in R6/2 mice following long-term PLX3397 treatment could at least in part be attributed to a decreased number of muscle macrophages. Collectively, these results suggest that targeting CSF-1R would be beneficial in HD. However, there are several shortcomings associated with the use of genetically modified small animal models to model the complex pathogenesis of HD. Small animal models fail to mimic the selective cortical and striatal neurodegeneration caused by ubiquitously expressed mHTT and striking neurodegeneration is not evident in transgenic mice expressing small N-terminal Htt repeats [126]. In addition, *HTT* may function differently in small and large animal brains. The HD monkey model exhibits clinical symptoms, including dystonia and chorea, and pathogenic features, including nuclear inclusions and neuropil aggregates, which are lacking in the transgenic mice generated using the same approach [127]. A novel huntingtin knock-in pig model [128] may serve as an important tool to validate the promising findings achieved using CSF-1R inhibitors in the rodent model of HD.

Other approaches using human materials such as biopsy- or iPSC-derived microglia and monocyte-derived microglia-like cells [129] may also help us to better understand the pathogenesis of HD in the future.

### Inhibiting the CSF-1R in ALS

ALS is a rapidly progressive neurodegenerative disorder with limited treatment options, caused by genetic and non-inheritable components that lead to significant loss of upper and lower motor neurons in the brainstem, motor cortex and spinal cord, and ending with respiratory muscle dysfunction [130]. ALS usually develops between the ages of 40 and 70. A variety of genes including superoxide dismutase 1 (*sod1*), transactive response DNA-binding protein 43 (*tdp-43*) and chromosome 9 open reading frame 72 (*c9orf72*) have been linked to the pathogenesis of ALS [131]. Transgenic rodent models expressing these genes can develop an ALS-like disease and SOD1^G93A^ mice are commonly used to explore ALS pathogenesis [132]. ALS is a heterogeneous illness involving oxidative stress, impaired autophagy, dysfunctional mitochondria and misfolding of proteins [133–135].

Although ALS is not initiated by inflammatory responses, neuroinflammation mediated by reactive glial cells and infiltrating leukocytes is increasingly recognized as a prominent pathological feature of ALS [131, 136, 137]. In support of this, widespread microglial activation as measured by PET is evident in ALS [138] and changes of microglial genes may occur before motor neuron damage [139]. Activated microglia have also been suggested to serve as a potential source of aberrant extracellular microRNAs contributing to neurodegeneration in ALS [140]. In addition, circulating monocytes from individuals with ALS are skewed towards a pro-inflammatory state [141]. Thus, efforts to reverse dysfunctional myeloid cells in ALS represent a potential therapeutic goal.

A pioneering study has indicated that treatment with the CSF-1R inhibitor GW2580 has beneficial effects in SOD1^G93A^ mice [142]. In this model, increased expression of CSF-1R and CSF-1, but not IL-34, was recorded in the spinal cord during pathogenesis [142]. GW2580 treatment through oral gavage starting from 8 weeks of age significantly depleted activated microglia and attenuated ALS-related motor deficits, as measured by rotarod testing and treadmill tests [142]. Strikingly, treatment with the CSF-1R inhibitor significantly increased the survival of SOD1^G93A^ mice, extending the maximal lifespan by 12% [142]. GW2580 treatment also reduced the numbers of circulating monocytes and their influx into the tibial nerve in SOD1^G93A^ mice, suggesting that peripheral immunity also plays a role in ALS [142]. Indeed, suppression of pro-oxidative function in the peripheral myeloid cells of SOD1^G93A^ mice (by genetic targeting of Nox2 or overexpression of wild-type human SOD1) reduced the proinflammatory activation of microglia, delayed symptoms and increased survival [143]. In summary, CSF-1R inhibition may slow the disease progression of SOD1^G93A^ mice by targeting both central and peripheral immunity.

However, the SOD1^G93A^ mouse model only recapitulates a small subpopulation of ALS patients, with > 90% patients developing sporadic disease [139]. Additional investigations are thus needed to validate the beneficial outcomes of CSF-1R inhibitor treatment in ALS. Importantly, an open-label phase 2 clinical trial investigating the safety and tolerability of the CSF-1R inhibitor BLZ945 in patients with ALS is ongoing (ClinicalTrials.gov, identifier: NCT04066244).

### Inhibiting the CSF-1R in MS

MS is a chronic demyelinating disease, with inflammation being the predominant neuropathological feature during the early course-relapsing–remitting disease phase [144]. It is well accepted that major differences exist between relapsing–remitting and primary progressive MS, but there are no clear clinical, radiological or biological boundaries of MS phenotype separation. Recently, a novel objective automatic classifier has been proposed using clinical information for assessing the MS phenotype, yielding a relatively high accuracy [145] that may be beneficial for further research. Progressive types of MS are characterized by irreversible accumulation of neurological disability which is mainly driven by neurodegeneration [146]. Individuals with primary progressive MS are usually diagnosed after the age of 40. Although it declines with age, neuroinflammation is still evident in some progressive MS individuals with clinical or radiological evidence of disease activity [147, 148]. Chronic activation of microglia contributes to neuroinflammation and neurodegeneration in MS [149]. In support of this, widespread abnormal glial activation was observed in progressive MS, as measured by ^18^F-PBR06 PET imaging [25, 150].

Levels of CSF-1R and its ligand, CSF-1, in CNS tissues are significantly increased in both preclinical mouse models and in MS patients [16]. Strikingly, selective inhibition of CSF-1R using a CNS-penetrant small molecule inhibitor, sCSF-1R_inh_, markedly attenuated disease severity, particularly during disease progression, in the preclinical models, mainly by reducing neuroinflammation, axonal degeneration and the production of proinflammatory cytokines [16]. Consistently, pharmacological depletion of microglia using another CSF-1R inhibitor (BLZ945) in the murine cuprizone model of demyelination effectively enhanced remyelination in a brain region-specific manner [50]. These beneficial effects on remyelination and disease recovery following the inhibition of CSF-1R have been reproduced in later studies using two other inhibitors (PLX3397 or PLX5622) [151, 152]. Thus, inhibiting CSF-1R in progressive MS populations during an early disease stage is an attractive treatment option.

## Potential side effects following CSF-1R inhibitor treatment

### Adverse effects in CNS development and homeostasis

Microglia play a well-documented role in regulating synapse development and connectivity [153]. Removing microglia using CSF-1R inhibitors during critical neurodevelopmental periods could cause potential side effects. For example, elimination of microglia using PLX5622 beginning at embryonic day 3.5 (E3.5) and ending at E15.5 increased the numbers of active cleaved Caspase 3^+^ apoptotic cells in the developing hypothalamus [154] and produced female-specific long-term behavioral alterations, with juvenile mice becoming hyperactive and adult mice exhibiting anxiolytic-like behavior [154]. Notably, mouse pups exposed to PLX5622 in utero also suffered from craniofacial and dental defects due to non-CNS effects on macrophages and osteoclasts [154].

CSF-1R inhibition might also be harmful during the early postnatal period. Two-week PLX3397 treatment starting at postnatal day 14 significantly increased dendritic spine density in the primary visual cortex, and changed spontaneous synaptic activity, subsequently disrupting cortical plasticity [155]. These results indicate that CSF-1R inhibitor treatment during development may alter functional connectivity in the CNS. In addition, treatment of mice with BLZ945 during the early postnatal period dramatically reduced the number of oligodendrocyte progenitors [5].

Importantly, detrimental effects of CSF-1R inhibition in adults have also been reported. Acute genetic microglial depletion in adult mice leads to neurodegeneration in the somatosensory system, associated with a type I interferon signature [156]. Transient PLX5622-mediated depletion of microglia in adult mice impaired the integration of adult-born granule cells in the olfactory bulb circuit and caused reduced odor-evoked responses [157] and ablation of microglia might cause seizures by influencing the activity of neurons [106]. Furthermore, PLX3397 or PLX5622 treatment reduces the numbers of PDGFRα^+^ or NG2^+^ oligodendrocyte progenitor cells*,* but not of mature oligodendrocyte cells [158]. Furthermore, administration of CSF-1R inhibitors to adult mice causes significant loss, not only of microglia, but also of ~ 60% of brain-resident perivascular macrophages [159].

In summary, potential side effects of CSF-1R inhibitor treatment should be carefully considered, particularly during organogenesis and neurodevelopment.

### Non-CNS effects

Administration of anti-CSF-1 antibody to mice during the first 60 days of life induces phenotypes similar to those reported in CSF-1- and CSF-1R-deficient mice, including decreased growth rate osteopetrosis associated with decreased osteoclast numbers and decreased macrophage densities in bone marrow, liver, dermis, synovium and kidney and decreased adipocyte size in the adipose tissue [160]. Administration of CSF-1R inhibitors to adult mice also affects monocyte maturation and causes the loss of several peripheral tissue macrophages including those present in the liver, spleen and sciatic nerves [64, 161]. Furthermore, both PLX5622 and PLX3397 significantly reduced numbers of circulating Gr1^low^ (Ly6C^−^) monocytes [125]. Consistent with its broader specificity, PLX3397 treatment at a dose of 400 mg/kg markedly altered the blood cell composition by decreasing the numbers of red blood cells, hemoglobin, platelets, and dendritic cells [94]. CSF-1R inhibitors also exert important effects on trabecular bone density in adult mice [162]. The potential peripheral consequences following CSF-1R inhibitors should be carefully considered.

Of disease and translational research relevance, another major concern would be the toxicity of long-term therapeutic administration of CSF-1R inhibitors. In 2019, Pexidartinib (PLX3397) was approved by the Food and Drug Administration (FDA) for the treatment of selected adult individuals with tenosynovial giant cell tumors (a connective tissue disease driven by CSF-1 in an autocrine manner) [163, 164]. The recommended oral dose is 400 mg twice per day in clinical practice. In a phase 3 clinical trial several adverse events following more than 20 weeks’ Pexidartinib treatment were reported [164], the most common of these being hair discoloration (67%) [163]. We and others have noted similar phenomena in preclinical mouse models treated with PLX3397 [69, 165]. It was suggested that changes of hair color might be attributed to the inhibition of C-KIT [163]. Importantly, Pexidartinib may also cause hepatotoxicity such as reversible raised levels of alanine aminotransferase (ALT) and aspartate aminotransferase (AST) due to effects on Kupffer cells, the resident macrophages in the liver [163]. All cases were reversible upon discontinuation of the drug without functional liver damage or structural damage to hepatocytes [54]. It is important to note that CSF-1R inhibitors are usually supplied via the diet in preclinical research settings [71]. It was shown that food intake (either high or low-fat) altered exposure as well as pharmacokinetics. Furthermore, it was suggested that these alterations could be alleviated by taking the drugs on an empty stomach or a few hours after a meal [166].

### Risk profiles

A study reporting that microglial depletion using PLX5622 in a mouse model of prion disease resulted in prion accumulation and acceleration of disease [167], raises concerns that CSF-1R inhibitors may exacerbate prion diseases, such as Creutzfeldt-Jakob disease (CJD), fatal familial insomnia (FFI) or Gerstmann-Sträussler-Scheinker disease (GSS).

CSF-1R inhibitors may also cause or exacerbate pathogenic infection of the CNS. Blocking CSF-1R selectively inhibited Th2 memory function in a mouse model of chronic asthma [168]. Other preclinical studies indicate deleterious effects of CSF-1R inhibition in viral infections. Infection of Theiler’s murine encephalomyelitis virus (TMEV) in C57BL/6J mice, a preclinical model of spontaneous recurrent seizures, is typically cleared within 14 days. However, TMEV infection in mice depleted of microglia following treatment with PLX5622 leads to fatal viral encephalitis, even after infection with low viral loads [169]. Similarly, in mice infected with a neurotropic strain of mouse hepatitis virus (a group 2 coronavirus), PLX5622 treatment delayed clearance of the virus and increased viral protein load in neurons [170] and herpes simplex virus 1 infection in mice in which the CSF-1R was conditionally deleted decreased survival rate and the ability to control the viral replication [171]. These results suggest that the use of CSF-1R inhibitors might suppress the antiviral response of the CNS [169, 170] and poses a challenge for clinical management of patients and care settings, particularly during the current COVID-19 pandemic [172].

## Concluding remarks

Despite promising results from preclinical studies with CSF-1R inhibitors, we are still facing the challenge of translating these findings into clinical practice. The failure of anti-inflammatory treatments involving global elimination of microglia in neurodegenerative diseases, suggests that dysfunctional microglia might need to be targeted in a spatio-temporally controlled manner. This approach would require investigation of their heterogeneity and functional plasticity in different stages of the disease. CSF-1R inhibitors were mostly tested in preclinical studies during a pre-symptomatic period, or even before onset. However, most patients with neurodegenerative diseases might not be identified until an advanced disease stage. From a clinical perspective, more investigations should be performed at distinct disease stages (e.g. early versus late) to provide convincing evidence of efficacy. CSF-1R inhibition should be attempted in relatively old (> 6 months of age) rather than young mice. Furthermore, potential off-targets effects need to be addressed, especially in studies involving the oral administration of CSF-1R inhibitors.

## Data Availability

Data sharing is not applicable to this article as no datasets were generated or analysed during the current study.

## Abbreviations

Aβ: Amyloid beta
AD: Alzheimer’s disease
ALS: Amyotrophic lateral sclerosis
CNS: Central nervous system
CSF-1R: Colony stimulating factor 1 receptor
C9ORF72: Chromosome 9 open reading frame 72
FDA: Food and drug administration
HD: Huntington’s disease
IC_50_: Half maximal inhibitory concentration
IL: Interleukin
JAK/STAT: Janus kinase/signal transducers and activators of transcription
MPTP: 1-Methyl-4-phenyl-1,2,3,6-tetrahydropyridine
MS: Multiple sclerosis
PD: Parkinson’s disease
PDGFR: Platelet-derived growth factor receptor
PET: Positron emission tomography
PI3K: Phosphatidylinositol 3'-kinase
PTP-ζ: Protein-tyrosine phosphatase-ζ
SOD1: Superoxide dismutase 1
TDP-43: Transactive response DNA-binding protein 43
TGF-α: Transforming growth factor alpha
TNFα: Tumor necrosis factor alpha
FFI: Fatal familial insomnia
GSS: Gerstmann-Sträussler-Scheinker disease
